# Temporarily Epigenetic Repression in Bergmann Glia Regulates the Migration of Granule Cells

**DOI:** 10.1002/advs.202003164

**Published:** 2021-03-22

**Authors:** Shaoxuan Chen, Kunkun Zhang, Boxin Zhang, Mengyun Jiang, Xue Zhang, Yi Guo, Yingying Yu, Tianyu Qin, Hongda Li, Qiang Chen, Zhiyu Cai, Site Luo, Yi Huang, Jin Hu, Wei Mo

**Affiliations:** ^1^ State Key Laboratory of Cellular Stress Biology The First Affiliated Hospital of Xiamen University School of Life Sciences Xiamen University Xiamen 361102 China; ^2^ The Department of Neuroscience School of Medicine Xiamen University Xiamen 361102 China; ^3^ Xiang'an Hospital of Xiamen University School of Medicine Xiamen 361102 China; ^4^ National Institute for Data Science in Health and Medicine Xiamen University Xiamen 361102 China; ^5^ Key Laboratory of Ministry of Education for Coast and Wetland Ecosystems College of the Environment and Ecology Xiamen University Xiamen 361102 China; ^6^ Department of Clinical Laboratory Fujian Provincial Hospital Fuzhou 350001 China; ^7^ Provincial Clinical College Fujian Medical University Fuzhou 350001 China

**Keywords:** Bergmann glia, BG development, ECM, GC migration, H3K9me3, Nfix, Setdb1

## Abstract

Forming tight interaction with both Purkinje and granule cells (GCs), Bergmann glia (BG) are essential for cerebellar morphogenesis and neuronal homeostasis. However, how BG act in this process is unclear without comprehensive transcriptome landscape of BG. Here, high temporal‐resolution investigation of transcriptomes with FACS‐sorted BG revealed the dynamic expression of genes within given functions and pathways enabled BG to assist neural migration and construct neuron‐glia network. It is found that the peak time of GCs migration (P7‐10) strikingly coincides with the downregulation of extracellular matrix (ECM) related genes, and the disruption of which by Setdb1 ablation at P7‐10 in BG leads to significant migration defect of GCs emphasizing the criticality of Nfix‐Setdb1 mediated H3K9me3 repressive complex for the precise regulation of GCs migration in vivo. Thus, BG's transcriptomic landscapes offer an insight into the mechanism by which BG are in depth integrated in cerebellar neural network.

## Graphical abstract

Functional cerebellum relays on the coordinated spatiotemporal interplay between neurons and glia. The remarkable down‐regulation of components of ECM is critical for GCs migration at very narrow time window (P7–P10), and transcription factor Nfix recruits Setdb1, a tri‐methyltransferase of H3K9 to load H3K9me3 modification on ECM genes that should be repressed in BG at P7–P10.

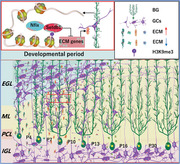

## 1 Introduction

Cerebellum is a well‐organized region of brain, which not only controls motor behavior but also regulates learning, cognition, and affective processes.^[^ ^1^, ^2^ ^]^ The mature cerebellar cortex consists of three layers: an inner granular layer (IGL), a Purkinje cell layer (PCL), and a molecular layer (ML);^[^ ^3^ ^]^ and the developing cortex has an additional external granular layer (EGL).^[^ ^4^ ^]^ There are mainly five types of neural cells in different layer of the cerebellum, including oligodendrocyte (OL), astrocyte, granule cell (GC), Purkinje cell (PC), and Bergmann glia (BG) .^[^ ^5^ ^]^ The precise location of these cells is critical for three‐layered laminar structure and foliation of the cerebellum in which BG plays a pivotal role in cerebellar morphogenesis.^[^ ^6^ ^]^ The malformation of cerebellum is related to various neurological disorders including ataxic, autism spectrum disorder (ASD), and so on.^[^ ^7^, ^8^, ^9^ ^]^

As a type of astroglia, BG are derived from radial glia cells and share some molecular signatures with astrocytes.^[^ ^10^ ^]^ In the other hand, BG is a special glia for their unique location and morphology. Located at the PCL, BG's cell bodies extend radial fibers through the ML to pial surface, connecting their endfeet to form the glia limitans.^[^ ^11^, ^12^, ^13^ ^]^ BG appear to sustain such unique morphology from early birth on; however, the functions of BG are variant over time to match continuously microenvironmental changes.^[^ ^13^, ^14^ ^]^ BG provide structural support for guiding the migration of newly differentiated GCs from the EGL to the IGL during first 2 weeks postnatally. The peak of GCs migration is at P7‐12, whose impairment results in disruptive morphology and paralyzed neural network of cerebellum.^[^ ^2^, ^15^, ^16^, ^17^ ^]^ After that, BGs are further integrated into neural circuit, playing critical roles in the information processing, structural integrity maintenance and synaptic connections.^[^ ^13^, ^18^ ^]^

The molecular mechanisms to regulate BG function are fragmented. Neither key transcriptional factors (TFs) nor epigenetic modifications are well studied in BG. Some signaling pathways, such as SHH, Notch, and Integrin‐linked kinase in BG are assistant for GCs migration.^[^ ^19^, ^20^, ^21^, ^22^ ^]^ Reports also show the influence of ECM (extracellular matrix)‐receptor interaction signaling on BG‐dependent GCs migration.^[^ ^23^, ^24^, ^25^ ^]^ Single cell sequencing of developing cerebellum yields a clear BG population; however, the features of it are obscure with very limited depth of transcriptome.^[^ ^26^ ^]^ More accurate and deeper omics analysis are necessary with purified BG.

In this study, we identified two mouse models to label BG and construct transcriptional landscape of BG from birth to adulthood. We defined the functions of BG with up‐regulated genes by multiple comparison (UGMC) at each stage of development. We found in surprise the progress of development relied on the dynamic expression of ECM‐related genes which functionally related with BG development and assisting of GCs migration. At the peak time of the cerebellar development (from P7 to P10), our data indicated that genes down‐regulated in this time window were required for GCs migration and exactly suppressed by Nfix‐Setdb1 repressive complex. Setdb1 is one of the main methyltransferases for the trimethylation of histone 3 lysine 9 (H3K9me3).^[^ ^27^, ^28^ ^]^ Mice with Setdb1 deleted at P7 in BG show the defect of GCs migration with abnormal upregulated genes that should have been inhibited by Nfix‐Setdb1 complex in wildtype (WT) BG. Together, we have systematically described the molecular characteristics of BG cells in each development stages, and further found that the transcriptional suppression by Nfix‐Setdb1 complex is critical for BG to assist the migration of GCs.

## 2 Results

### 2.1 Identifying Mouse Models for the Omics Study of Bergmann Glia

**Figure 1:**
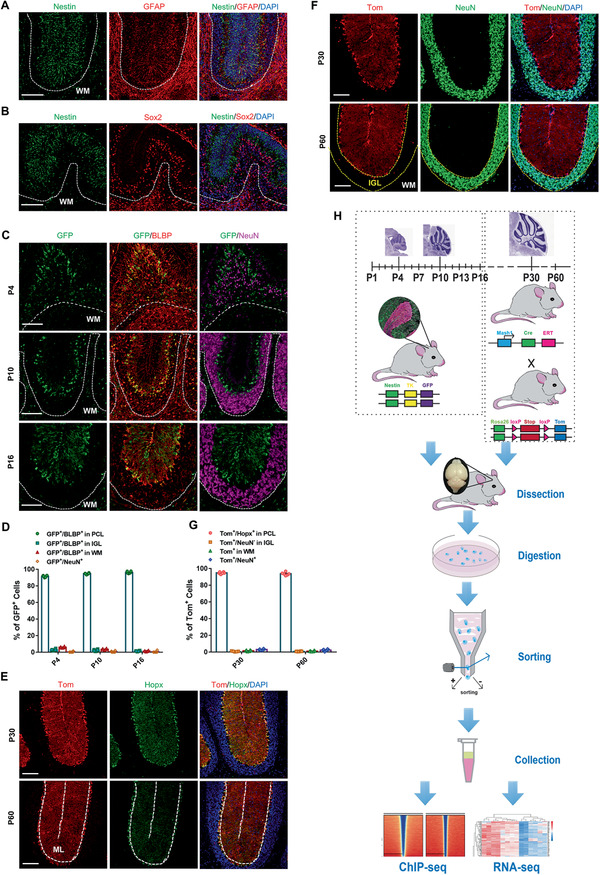
Identification of mouse models significantly labeling Bergmann glia. A,B) Co‐immunostaining of Nestin with A) GFAP or B) Sox2 in P7 cerebella, the white matter (WM) was indicated. C,D) Co‐immunostaining of GFP with BLBP or NeuN (C) in Nes^GFP^mouse at the time points as indicated (the WM was indicated). The ratio of different kinds of GFP positive cells at P4, P10, P16 were quantified (D), in which BLBP^+^ cells in PCL represent BG; BLBP^+^ cells in IGL and WM are assumed to be astrocytes; and NeuN^+^ cells refer to GCs. *n* = 6 sections from 3 mice per time point. E–G) Co‐immunostaining of tdTomato (Tom) with Hopx (E) or NeuN (F) inMash1^iTom^ mice at P30 and P60. The ratio of different kinds of Tom positive cells at P30, P60 were quantified (G), in which Hopx^+^ cells in PCL represent BG, NeuN^+^ cells refer to GCs, Tom^+^ cells in WM and Tom^+^/NeuN^−^ cells in IGL are assumed to be OLs, astrocytes or other type of cells. *n* = 6 sections from 3 mice per time point. H) The experimental strategy and workflow for BG isolation and next generation sequencing. All the quantification data are presented as mean ± SEM. Scale bars, 100 µm. See also Figure S1, Supporting Information.

General astro‐glia makers like GFAP and BLBP label both BG and astrocytes (**Figure** **1**A,C, middle panel) which make it infeasible to isolate BG by those well‐known markers.^[^ ^29^, ^30^ ^]^ Resembling to radial glia, BG express some of neural stem cell markers like Nestin, Sox2, Sox9, and so on, and Nestin enriched in BG (Figure 1A,B and data not show).^[^ ^31^, ^32^ ^]^ Nestin‐∆TK‐IRES‐GFP (Nes^GFP^) mice have GFP expression under control of rat Nestin promoter with 2nd intron restricting GFP signal in neural cells.^[^ ^33^ ^]^ In the first 2 weeks postnatally, overwhelming majority of GFP signals was located in BG (Figure 1C,D) with a very tiny portion in OLs (Figure S1A,C, Supporting Information) but not in GCs (Figure 1C,D) nor PCs (Figure S1B,C, Supporting Information). BG at P1, P4, P7, P10, P13, and P16 were sorted by GFP^+^ signals and other cells without GFP signals were gated out (Figure S1H, Supporting Information). Given that GFP signals went dimly in brain of Nes^GFP^ mice after 2 weeks postnatally, another mouse model was constructed to label BG since young adult stage. Mash1 Cre‐ERT2, R26‐LSL‐tdTomato (Mash1^iTom^) mice have bright red signals under control of Mash1 promoter expressing in BG but very few in other cells upon Tamoxifen (TMX) induction at P30 and P60, respectively (Figure 1E–G and Figure S1D–G, Supporting Information).^[^ ^34^, ^35^ ^]^ Then deep profiling for BG transcriptome was performed using sorted BG (Figure 1H and Figure S1H,I, Supporting Information) at 8 time points (P1, P4, P7, P10, P13, P16, P30, and P60).

### 2.2 Confirming the Reliability of Bergmann Glia Expression Profile and Obtaining its New Marker Genes

**Figure 2:**
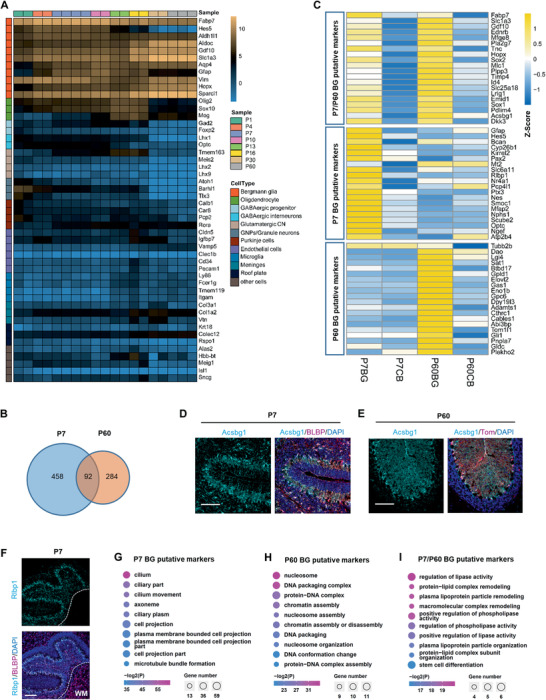
Confirming the reliability of Bergmann glia expression profile and obtaining its new marker genes. A) Heatmap to show the expression of marker genes of different kinds of cerebella cells. B) Venn Diagram to show the number of BG putative marker genes for juvenile (P7), young adult (P60), and for both juvenile and young adult. C) Heatmap to exhibit the expression of top 20 unique putative marker genes of juvenile (P7) or young adult (P60) BG and 20 common putative marker genes of juvenile (P7) and young adult (P60) BG. D–F) Immunofluorescence staining to verify the expression of BG new marker genes. D) Co‐staining of a new BG lineage marker Acsbg1 with a well‐established marker BLBP at P7 (The WM was indicated) or E) with Tom (expressed in Mash1^iTom^ mice) at P60. F) Co‐staining of a new marker Rlbp1 unique to juvenile BG with BLBP. Scale bars, 100 µm. G–I) GO analysis on putative marker genes of BG in the juvenile and/or young adult periods as indicated. Data display top 10 enriched GO terms ranked by p values. Color indicates p values for GO term enrichment and circle size indicates the number of enriched genes for each GO term.

Since BG's transcriptome has not been well documented ever, we compared our dynamic transcriptomic profiles with well‐accepted lineage markers of variant nerve cells by heatmap analysis which confirmed the significant enrichment of BG genes (**Figure** **2**A).^[^ ^36^ ^]^ P7 and P60 represented the peak and the termination of cerebellar development after birth, respectively. Compared the BG profile with that of whole cerebellum at the same time point, 458 and 284 genes are upregulated eight times or more in P7 and P60 BG separately (Figure 2B). These remarkable genes covered majority of reported BG marker genes.^[^ ^37^ ^]^ Thus, 92 genes upregulated eight times in both P7 and P60 were defined as BG lineage marker genes; and 270 or 156 genes only upregulated eight times in either P7 or P60 were named as stage‐specific BG marker genes (Figure 2B and Table S1, Supporting Information). Top 20 genes of each three clusters were shown as heatmap emerging a broader list of marker genes in BG (Figure 2C and Table S1, Supporting Information). We verified the expression of some new marker genes in developmental or mature BG by immunostaining or public ISH database from Allen lab or Single Cell Sequencing data presented in the “Cell Seek” website, further authenticating the accuracy of transcriptomes of BG (Figure 2D–F and Figure S2A–J, Supporting Information).^[^ ^36^ ^]^ To obtain an overview of these marker genes, Gene ontology (GO) analysis was performed (Figure 2G–I). For P7, top 10 GO terms pointed to the cilium and projection of BG fibers, which was very possible to support the GCs migration (Figure 2G). In contrast, the top 10 terms of P60 were totally different with that of P7, which showed signatures of chromatin remodeling (Figure 2H). We speculate that it may reflect the termination of violent changes in transcriptome. The unique signatures of BG compared with other cells in cerebellum were related with lipid metabolism (Figure 2I) which is worth to be further investigated.

### 2.3 Functional Analysis for UGMC of Bergmann Glia

To characterize more comprehensive features of BG and define the functions of BG at each stage, differentially expressed genes (DEGs, fold change ≥ 2) were analyzed (Figure S3A, Supporting Information). Genes highly expressed both in P7 and P60 were limited in number (Figure S3B, Supporting Information), which exhibited the both characteristics of fibers projection (Figure S3C, Supporting Information) and lipid metabolism (Figure S3D, Supporting Information).^[^ ^38^, ^39^ ^]^ Although in general P7 BG present a developmental signature (Figure S3E, Supporting Information), top 10 pathways enriched from P7 BG UGMC were shown to be related with GCs migration by “ECM‐receptor interaction”, “focal adhesion”, etc.^[^ ^23^, ^24^, ^40^ ^]^ (Figure S3F, Supporting Information). P60 BG UGMC significantly enriched metabolic pathways and showed intensive communication with neural microenvironment via extracellular transport by “extracellular vesicle, organelle, exosome” which were top 3 terms of GO (Figure S3B,G,H, Supporting Information). Together, the transcriptional landscape we obtained can well reflect BG's characteristics and functions in juvenile and adult, showing how BG supported the building of nerve network in cerebellum.

### 2.4 Determine the Milestones for Bergmann Glial Postnatally

**Figure 3:**
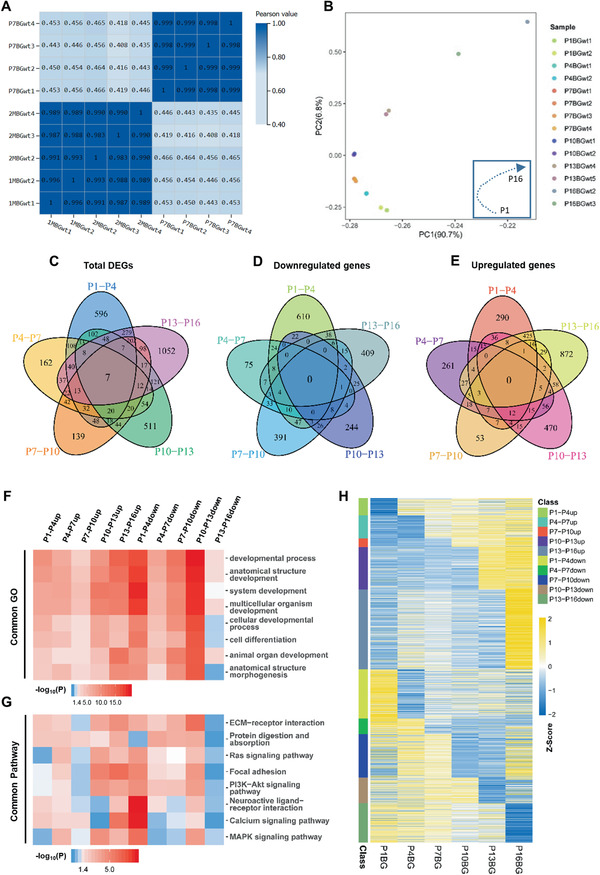
Exploring the intrinsic impetus to promote the developmental process of BG. A) Correlation heat map to analyze the similarity of BG transcripts between the juvenile stage (P7) and young adult stages (P30 and P60) with 2–4 repetitions at each time point. The color represents the correlation coefficient (the darker color represents the higher correlation). B) PCA shows the similarity of BG transcripts from P1 to P16 with 2–4 repetitions at each time point. C–E) Venn diagram to analyze the intersection of five adjacent groups (P1–P4, P4–P7, P7–P10, P10–P13, P13–P16) of DEGs. The intersections of C) total DEGs, D) downregulated genes, and E) upregulated genes of each group were shown. F,G) GO and Pathway analysis were performed on up or down regulated genes of the five adjacent stages of BG within P1 to P16, and the top 8 of most common GO (F) or pathway (G) terms were shown in the heatmaps. H) Heatmap to display the successive expression patterns of all up or down regulated genes in (C) from P1 to P16.

To determine the termination of BG develop in molecular level, all the profiles at variant time points were compared with that of developing (P7) and developed (P60) BG, showing that 1‐month BG profile was almost identical with that of 2 months (**Figure** **3**A), whereas 1‐month and P16 BG showed very distinguished pattern (Figure S4A, Supporting Information). This indicated BG profile keep stable since P30. The characteristics of P16 transcriptome were mixed with that of P7 and P60. The top 10 GO terms of downregulated genes from P16 to 1 month were genes of development or fiber projection (Figure S4B, Supporting Information), which were similar with that of P7 BG UGMC (Figure S3E, Supporting Information); and the top 10 GO terms of up‐regulation genes (Figure S4C, Supporting Information) were like that of UGMC in P60 BG (Figure S3G, Supporting Information). All of these indicate at molecular level, BG are mature at P30. A feature for the termination of development is significant downregulation of the cell cycle related genes which was the most noteworthy feature of down‐regulated DEGs during P13‐16 (Figures S4D,E, Supporting Information). In addition, we also observed the changes of genes for fiber projection during P13‐16 (Figure S4F, Supporting Information). All those evidences above suggest a transient state of pre‐mature BG at P13‐16. According to the expression changes of BG characteristic genes at different time points, the BG (P1–P60) can be divided into three distinct periods: developmental period, transition period, and mature period (Figure S4G, Supporting Information).

### 2.5 Exploring the Intrinsic Impetus to Promote the Developmental Process of Bergmann Glia

Then we focused on the dynamic changes of transcriptome in the developmental period. As expected, principal component analysis (PCA) showed the developmental changes in transcriptome continuously during first 2 weeks postnatally (Figure 3B). The overlap of DEGs in each developmental stage was very rare (Figure 3C–E); but in surprise, GO and Pathway analysis of each DEGs (Figure S4H–O, Supporting Information) showed very similar terms in entire developmental period (P1‐4, P4‐7, P7‐10, P10‐13). GOs pointed to development and fiber projection; and pathways pointed to GC migration, which was similar with terms of P7 in Figure S3E,F, Supporting Information. No doubt, common GO and pathway analysis for up/down DEGs in entire developmental period (P1‐13) (Figure 3F,G) were almost identical with those of P7 UGMC (Figure S3E,F, Supporting Information), which emphasized again BG's common characteristics and functions in first 2 weeks postnatally were assisting GC migration. In contrast, the terms showed obvious divergence in the down‐regulated genes in the transient period (P13‐16). BG in different developmental stages with distinct transcription profile processing the similar ability with neural migration indicates the change of genes for fiber projection and GCs migration is the intrinsic impetus promotes the BG program in developmental period. Further heat map analysis revealed these genes were only temporally changed expression in certain developmental stages (Figure 3H). Together, our data showed the dynamic of genes with given functions and pathways through which BG were continuously adjusted to adapt the requirement of neural migration in different developmental stages.

### 2.6 Setdb1 Cooperating with Nfix Act as a Candidate Negative Regulator in the Development of Bergmann Glia from P7 to P10

**Figure 4:**
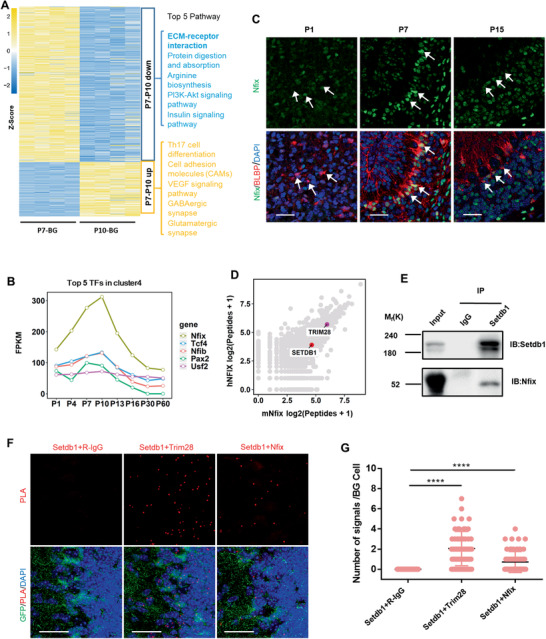
Identification of Nfix‐Setdb1 repressive complex for transcriptional repression in BG from P7 to P10. A) Heatmap suggests more genes were repressed rather than upregulated in BG during P7 to P10. The top 5 pathways (ranked by *p* values) were enriched. B) Time course for genes that most highly express in P7–P10 BG (related to cluster 4 of Figure S5A, Supporting Information), the top5 transcription factors ranked by FPKM are shown in this line chart. C) Co‐staining of Nfix and BLBP for the dynamic expression of Nfix protein in BG at different time points as indicated. D) Scatter plots shows proteins binding to hNFIX or mNfix by MS. E) Co‐immunoprecipitation of endogenous Nfix with Setdb1 from P8 sorted BG. F,G) PLA detects the endogenous binding of Setdb1 with Nfix in P9 cerebella of Nes^GFP^ mice (F). The binding of Setdb1 with Trim28 are used as positive control. The red dot signals represent protein binding, and the number of dots in BG cells (G) were counted, two‐tailed unpaired Student's *t*‐test, ****P < 0.0001, *n* = 60 BG cells per group. All the quantification data are presented as mean ± SEM. Scale bars, 30 µm.

To explore the mechanism by which the dynamic was sophistically controlled during developmental period, we focused on P7‐10 when BG mediated mass migration of GCs from EGL to IGL. Pathways like the “ECM‐receptor interaction pathway” for GC migration were downregulated; and interestingly, the down‐regulated genes were dominant at this short time window indicating that transcriptional repression is critical for BG development and GCs migration (**Figure** **4**A).

TFs are the major determinants of transcriptional regulation, and TFs with highest expression level at P7‐10 were most likely the master regulators for transcriptional repression in this stage. We divided all genes into 12 clusters by temporal expression patterns and found genes in cluster 4 had the highest expression level at P7‐10 (Figure S5A, Supporting Information). Ranking the TFs by FPKM in this cluster from high to low, the top1 TF, Nfix was chosen for following study (Figure 4B). Although Nfix was reported to express in GC, the expression of Nfix in P7 BG is higher than that of Nfix in P7 GC.^[^ ^41^ ^]^ The protein level of Nfix confirmed by immunofluorescence in BG was shown same temporal pattern with that of RNA (Figure 4C).

The transcriptional repressor complex is composed with TFs and epigenetic regulators. To uncover the epigenetic modifiers in Nfix‐mediated complex, mass spectrometry (MS) were performed using mouse and human NFIX as a bait, respectively. Both of them unveiled an intensive binding with Setdb1 and its coordinator Trim28 (Figure 4D). Both Setdb1 and Trim28 were expressed in BG (Figure S5B,C, Supporting Information). The interaction between Nfix and Setdb1 was confirmed by immunoprecipitation (IP) (Figure 4E and Figure S5D,E, Supporting Information). In addition, the in situ binding of Setdb1 with Nfix in BG was identified by PLA (Figure 4F,G). Thus, Nfix‐Setdb1 could compose a transcriptional repressive complex to depress genes at P7‐10 in BG, which could be indispensable for GC migration and function of cerebellum.

### 2.7 Setdb1 Deficiency of Bergmann Glia Results in Abnormal Localization of Cerebellar Cells

**Figure 5:**
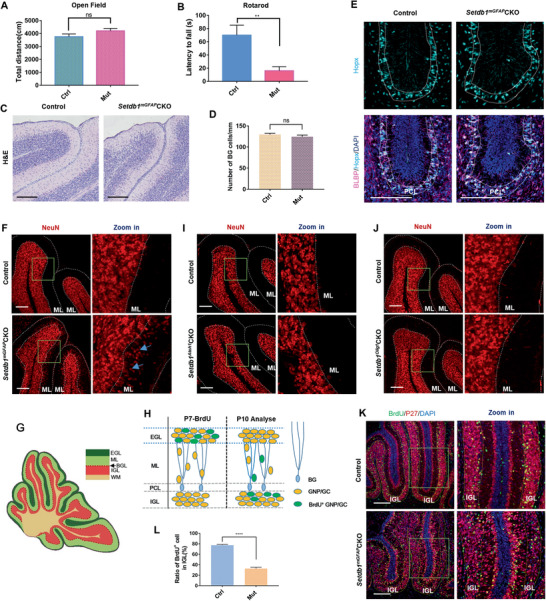
Setdb1 deficiency of Bergmann glia restrains GCs migration. A,B) Behavior tests were performed on control (Ctrl) and Setdb1^mGFAP^CKO (Mut) mice at P30 to detect its motor ability (A) with Open Field test, and balance ability (B) with Rotarod test, *n* = 15 for control and *n* = 14 for mutant mice. ns, no significance, **P = 0.0034. C) Hematoxylin and eosin (H&E) staining shows the morphology of the control and Setdb1^mGFAP^CKO cerebella at P15. D,E) Immunostaining for Hopx and BLBP (BG markers) in the control and Setdb1^mGFAP^CKO cerebella at P7 (the cell bodies of BG were localized in the PCL between the white line) (E), and the density for cell bodies of BG in PCL was quantified (D). ns, no significance, *n* = 16 sections from 4 mice for each group. F) NeuN staining shows the mature GCs in the control and Setdb1^mGFAP^CKO cerebella at P15. Arrows point to the ectopic cell mass in the ML in mutant mice. G) Diagram of different layers in Cerebellum. H) Diagram of BrdU pulse‐chase assay. Injection of BrdU at P7 to label proliferating GNPs within EGL. 3 days later, differentiated GNPs (BrdU^+^ GCs) are migrating to IGL through ML. I,J), NeuN staining shows the mature GCs in the I) Setdb1^Atoh1^CKO or J) Setdb1^Olig1^CKO cerebella and their controls at P15. K,L) P27/BrdU staining of cerebellar sections from P10 control and Setdb1^mGFAP^CKO mice that injected with BrdU at P7 (K). The ratio of BrdU^+^ cells in IGL (L) was shown, *n* = 11 for control and *n* = 7 for mutant mice, ****P < 0.0001. All the quantification data are presented as mean ± SEM, two‐tailed unpaired Student's *t*‐test. Scale bars, 150 µm.

To measure the importance of Nfix‐Setdb1 complex, Setdb1 was deleted in BG using mGFAP‐Cre, Setdb1^flox/flox^ (Setdb1^mGFAP^CKO) mice.^[^ ^42^, ^43^ ^]^ mGFAP‐Cre targets all nerve cells except for PCs in cerebellum (Figure S6A–F, Supporting Information), and thus Setdb1 can be successfully knocked out in BG (Figure S6B,F, Supporting Information). Setdb1^mGFAP^CKO mice born at Mendelian ratio with normal life span (data not shown). The mobility of mutant mice was comparable with that of control mice (**Figure** **5**A), but poor balance of Setdb1^mGFAP^CKO mice was observed revealing the disability of cerebellum (Figure 5B). Cerebellar anatomy of P15 Setdb1^mGFAP^CKO mice exhibited disordered cerebellum with obscure boundaries of IGL (Figure 5C). The number and morphology of BG were not significantly affected by Setdb1 deletion in P7 (Figure 5D,E and Figure S6G, Supporting Information). Not targeted by mGFAP‐Cre, the density of PCs was not affected (data not shown). In contrast, more mature GCs were retained in P15 ML (Figure 5F) and even 1‐month ML (Figure S6H, Supporting Information), suggesting the migration defect of GCs in Setdb1^mGFAP^CKO mice. Large number of granule neuron precursors (GNPs) start differentiation and migrate through ECL to IGL from P7 (Figure 5G,H). To exclude the dysfunction of GNPs by Setdb1 deletion, one‐day BrdU pulse‐chase assay was performed in Setdb1^mGFAP^CKO. The ratio of differentiated GNPs (BrdU^+^/p27^+^ cells) was not altered in Setdb1^mGFAP^CKO (Figure S6I–K, Supporting Information). In addition, Setdb1^Atoh1^CKO mice with deleted Setdb1 in GNPs and GCs (Figure S7A–E, Supporting Information) have normal GCs localization (Figure 5I) with intact BG (Figure S7F–H, Supporting Information).^[^ ^44^ ^]^ Those data indicate that the migration defect of GCs in Setdb1^mGFAP^CKO mice was not related with GCs themselves. Normal GCs localization (Figure 5J) with intact BG (Figure S7O–Q, Supporting Information) were also observed in Setdb1^Olig1^CKO mice which could exclude the influence from OLs, PCs, and astrocytes (Figure S7I–N, Supporting Information).^[^ ^45^ ^]^ To confirm the GCs migration defect in Setdb1^mGFAP^CKO mice, 3‐day BrdU pulse‐chase assay was performed. Nearly 80% of GNPs at P7 were migrated out of EGL and located in IGL at P10 in control mice. In contrast, less than 30% of GNPs at P7 reached IGL at P10 in Setdb1^mGFAP^CKO mice indicating that the migration of GCs was blunted when Setdb1‐composed repressive complex was absent in BG (Figure 5H,K,L).

### 2.8 Nfix‐Setdb1 Transcriptional Inhibitory Complex in BG Direct Affects Cerebellar GC Migration and Localization

**Figure 6:**
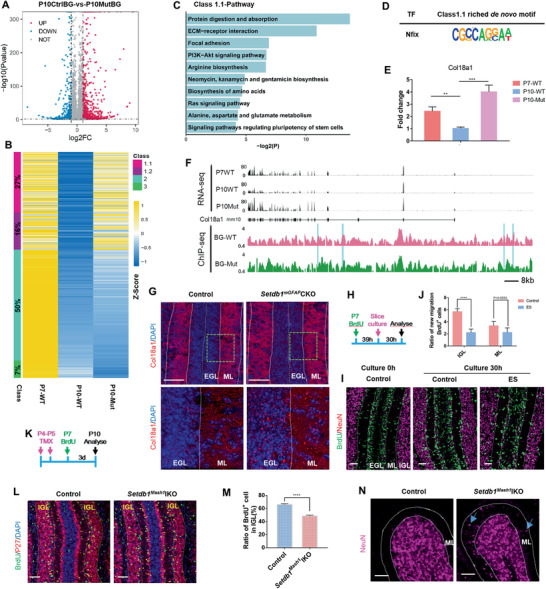
Nfix‐Setdb1 transcriptional inhibitory complex targets genes that downregulated for GC migration during P7–P10. A) Volcano plot shows DEGs of control (Ctrl) BG versus P10 mutant (Mut) BG. The control BG were sorted from Nes^GFP^ mice and the mutant BG were sorted from Setdb1^mGFAP^CKO‐Nes^GFP^ mice. B) The left two columns represent genes that are downregulated in BG during P7–P10. After Setdb1 deletion (the right column), the inhibition of those genes could be reversed (class 1.1 and 1.2), sustained (class 2) or enhanced (class 3). C) Representative top pathway terms enriched in transcripts from class 1.1 which were abnormal regulated genes by loss of Setdb1‐mediated H3K9me3‐binding sites in Setdb1^mGFAP^CKO BG. D) The de novo Nfix binding motif identified by HOMER derived from class 1.1 genes. E) The up‐regulation of Col18a1 in P10 Setdb1^mGFAP^CKO BG were confirmed by quantitative real‐time PCR, *n* = 6 independent experiments. F) Genome browser view of genetic and epigenetic landscapes surrounding the Col18a1 gene locus. Upper panel is an RNA‐seq peak visualization of gene expression from P7 WT, P10 WT, and P10 Mut. Lower panel shows H3K9me3 ChIP‐seq in P10 WT BG and P10 Mut BG. The specific H3K9me3 peaks with de novo Nfix motif in P10 WT BG were highlight in blue. G) Col18a1 staining shows the extracellular signals of Col18a1 in the control and Setdb1^mGFAP^CKO cerebella at P10. H–J) Using the ex vivo mouse CSC system to test for the effect on GCs migration with or without adding ES peptides which are 20 kDa C‐terminal cleavage product of Col18a1. The experiments were performed as (H). Cerebellar slices were stained for NeuN/BrdU (I). Initial migration ratio of GC (BrdU^+^ cells in IGL or ML / Total BrdU^+^ cells) was quantified from left panel of Figure 6I. The final migration ratio of GC (BrdU^+^ cells in IGL or ML / Total BrdU^+^ cells) was quantified from Figure 6I, middle and right panel, respectively. New migration ratio of GC = Final migration ratio − Initial migration ratio (J), *n* = 20 sections from 4 independent CSC for each group. K) Workflow of TMX administration in Setdb1^Mash1^IKO mice and BrdU pulse‐chase assay. L,M) Cerebellar sections from P10 control and Setdb1^Mash1^IKO mice were stained for P27/BrdU (L). The ratio of BrdU^+^ cells in the IGL was quantified, *n* = 24 sections from 3 mice for each group. 72 h after BrdU‐pulse (M). N) NeuN staining for control and Setdb1^Mash1^IKO cerebella at P15, arrows point to the ectopic cell mass in the ML. All the quantification data are presented as mean ± SEM, two‐tailed unpaired Student's *t*‐test, **P < 0.01, ***P < 0.001, ****P < 0.0001. Scale bars, 50 µm

Then the genes changed upon Setdb1 deletion in BG were assessed by RNA‐seq, which revealed 276 genes downregulated and 650 genes upregulated (fold change ≥ 2) in P10 (**Figure** **6**A). Considering the repressive complex was missed in mutant BG, we paid more attention to the genes that should be decreased during P7‐10. 43% of genes (class 1.1+1.2) that should downregulate at this stage were upregulated in mutant BG (Figure 6B). To define the genes that directly regulated by the repressive complex, ChIP‐seq was performed. Setdb1‐mediated H3K9me3 binding sites were defined as the H3K9me3 peaks present in WT BG but absent in mutant BG (Figure S8A, Supporting Information). 62.8% (class 1.1) of abnormal increased genes (class 1.1+1.2) in mutant BG were directly regulated by the H3K9me3 modification mediated by Setdb1 (Figure 6B). The top5 pathways enriched from the genes in class 1.1 (Figure 6C) were very similar with that of the genes which should have been down regulated during P7‐10 (Figure 4A). Notably, the top1 reduced pathway “ECM‐receptor interaction” in this stage (Figure 4A) was ranked No. 2 pathway in genes class 1.1 (Figure 6C). Importantly, Nfix motif was significantly enriched (Figure 6D) among them (class 1.1 genes), indicating the Nfix‐Setdb1 complex dominates the genes repression in BG for GCs migration. Regarding the role of “ECM‐receptor interaction” pathway in BG for GCs migration (Figure 3G,A,C and Figures S3F and S4J, Supporting Information), Col18a1, an ECM gene with several Nfix binding sites (Figure 6F) were picked up for validation. Col18a1 was also reported to regulate neuron migration.^[^ ^46^ ^]^ The expression of Col18a1 was repressed in WT BG during P7‐10 but increased in mutant BG (Figure 6E,F), due to lower H3K9me3 deposition (Figure 6F) the upregulation of Col18a1 in protein level was also observed (Figure 6G). To evaluate the effect of Col18a1 on GCs migration, ex vivo GCs migration assay was performed using cerebellar slice culture (CSC) system (Figure 6H, see the Experimental Section). To exclude the effect of Col18a1 on GCs proliferation, the cerebellar slice were obtained from pups with long (39 h) BrdU chase to make sure majority of BrdU^+^ GCs exited cell cycle in EGL (Figure S8B,C, Supporting Information). The ratio of migrated BrdU^+^ GCs at this time point was recorded as “Initial migration BrdU^+^ cells” (Figure 6I, left panel). Then the GCs migration in slice under endostatin (ES) peptides (20 kDa C‐terminal cleavage product of Col18a1) treatment were measured. These results (Figure 6I,J) provide the direct evidence of Col18a1 negatively regulating GCs migration. Together, Nfix‐guided and Setdb1‐catalyzed H3K9me3 modification declines the genes should have been repressed during P7‐10 for GCs migration.

To validate the necessarily of Nfix‐Setdb1 complex for GCs migration at P7‐10 window, we constructed Mash1 Cre‐ERT2, Setdb1^flox/flox^ (Setdb1^Mash1^IKO) mice. Setdb1 was deleted in BG (95.2%) at P7 by TMX administration at P4 and P5 (Figure S8D–K, Supporting Information). Although 98.4% of GNPs and 17.6% GCs were targeted by Cre activity (Figure S8H,J, Supporting Information), they had no major effects on GCs migration, which had been proved above (Figure 5I). This acute deletion of Setdb1 did not change the number and morphology of P7 BG (Figure S8L–N, Supporting Information). GNPs were recorded by BrdU at P7 which was the time point when Setdb1 was gone in BG. The number of BrdU^+^ GNPs in EGL and BrdU^+^/p27^+^ differentiating GCs were all identical indicating that neither proliferation nor differentiation of GNPs are affected in Setdb1^Mash1^IKO BG (Figure S8D,O–Q, Supporting Information). In contrast, the migration of GCs was blocked in some extent as less differentiated GCs in Setdb1^Mash1^IKO mice reached IGL at P10 (Figure 6K–M). In addition, the retention of mature GCs at ML was observed in P15 Setdb1^Mash1^IKO cerebellum (Figure 6N), indicating the transcriptional repressor composed by Nfix and Setdb1 in BG was required for GCs migration at P7‐10 stage.

## 3 Discussion

The research of neurons, glia, and stem/ progenitor cells in brain becomes more precise, comprehensive and systematic with the wider use of omics in recent year.^[^ ^47^ ^]^ Previous study took 6 individual BG from P6 and P30, respectively, for microarray and revealed 560 DEGs.^[^ ^26^ ^]^ Jian Peng et al. analyzed transcriptome profiles of 21 119 single cells of the postnatal mouse cerebellum but did not identify a separated BG population from astrocytes.^[^ ^48^ ^]^ scRNA‐Seq of 39 245 single cells mixed from E10‐ P10 mouse cerebella sequence yielded 5 glia populations, 2 of which were identified as BG.^[^ ^36^ ^]^ However, the molecular trajectory analysis for pseudo‐time of BG development and differentiation was not provided. In this manuscript, two mouse models were established/identified to significantly enrichment of BG in developmental and mature stage, respectively (Figure 1 and Figure S1, Supporting Information). In this study, whole genome transcriptomes obtained in terms of BG at multiple critical time points sorting from these two models were performed, shedding light on the molecular pictures of BG with temporal variation from birth to adult.

Although maintaining their morphology and localization since P7, BG have different functions in cerebellum postnatally which was truly represented by our RNA‐seq.^[^ ^13^, ^18^ ^]^ We identified the BG symbol genes for juvenile and/or adult (Figure 2A). We found the general functions of DEGs for each adjacent stage in juvenile are closely relevant with the migration of GCs (Figure 3 and Figure S4, Supporting Information). At P7‐10 when the peak of GCs migration starts, Nfix‐Setdb1 mediated transcriptional repression should be absolutely necessary suggested by bioinformatic analysis and validated in vivo using mouse model (Figures 4, 5, 6). Mice with disrupted repressive complex by genetic ablation of Setdb1 has balance disorder, cerebellar deformity, and GCs migration defect (Figure 5). Consistently, the addition of ES peptides for the cerebella slice culture also affects the GCs migration (Figure 6). Thus, the conclusion from bioinformatic analysis downregulation of genes for ECM‐receptor interaction in BG is required for the GCs migration has been well supported by the vivo and ex vivo data.

Previous studies showed that H3K9me3 modification is essential for embryonic development, and the construction of the central nerve system.^[^ ^43^, ^49^ ^]^ Loss of Setdb1 in embryonic neural stem cells by Nestin‐Cre led to apoptosis in embryonic cortex and mice mortality after birth.^[^ ^50^ ^]^ Our recent work shown Setdb1 safeguards the genome stability in intestinal stem cells. Setdb1 deficiency causes the activation of endogenous retrovirus elements which triggers ZBP1‐mediated necroptosis for bowel inflammation.^[^ ^28^ ^]^ Setdb1^mGFAP^CKO mice are able to keep alive with normal motor ability (Figure 5A and data not shown). Neither cell death nor inflammation was detected in Setdb1 deleted BG (Figure S9A–C, Supporting Information). Given mGFAP‐Cre target BG, GNPs, and OLs, Setdb1^Atoh1^CKO and Setdb1^olig1^CKO mice were examined and found no GCs migration defect at all (Figure S7, Supporting Information), clearly demonstrating Setdb1 null BG affected GCs migration with a non‐autonomous manner in Setdb1^mGFAP^CKO mice.

Setdb1 mutations have been found in a few autistic patients, which suggests Setdb1 might be related to the pathogenesis of ASD.^[^ ^51^ ^]^ In addition, some studies also reported the role of Setdb1 in mood regulation and the pathogenesis of schizophrenia.^[^ ^52^, ^53^ ^]^ In the Open Field test, we noticed that Setdb1^mGFAP^CKO mice always walked around the edge of the box repeatedly (data not shown). Such anxiety or stereotypical behavior in Setdb1^mGFAP^CKO mice implies that the role of Setdb1 in other nerve cells needs to be further studied.

## 4 Experimental Section

##### Mice

All mice used regardless of gender were housed in a vivarium with 12 h light/dark cycle and free access to water and food at the core animal facility of Xiamen University. All experimental procedures were approved by the Institutional Animal Care and Use Committee at Xiamen University. To generate Mash1^iTom^ mice, Mash1 Cre‐ERT2 mice and R26‐LSL‐tdTomato mice were crossed.^[^ ^34^, ^35^ ^]^ Similar way to get Olig1^Tom^ mice, which were using Olig1‐Cre mice crossed with R26‐LSL‐tdTomato mice.^[^ ^45^ ^]^ As for mGFAP‐Cre, R26R‐LacZ mice, the R26R‐LacZ mice, and mGFAP‐Cre mice were mated. Setdb1^flox/flox^ mouse strains were obtained from Dr. Yan Jiang.^[^ ^42^, ^43^, ^54^ ^]^ Setdb1 CKO (conditional knock out) and one Setdb1^Mash1^IKO (induced knock out) mouse lines were generated via crossing Setdb1^flox/flox^ with mGFAP‐Cre, Atoh1‐Cre, Olig1‐Cre, or Mash1 Cre‐ERT2 mice, respectively.^[^ ^44^ ^]^ To generate Setdb1^flox/flox^‐Nes^GFP^ mice, Setdb1^flox/flox^ mice were mated with Nes^GFP^mice, they further being crossed with mGFAP‐Cre to produce Setdb1^mGFAP^CKO‐Nes^GFP^ mice.^[^ ^33^ ^]^

##### Isolation of Bergmann Glia From Cerebella

Brains from Nes^GFP^ or Mash1^iTom^ mice were rapidly put into ice cold phosphate‐buffered saline (PBS) and dissected under a dissecting microscope. Cerebella were then fully cut into smaller pieces and incubated in papain solution (Ca^2+^‐ and Mg^2+^‐free HBSS, Hank's Balanced Salt Solution, within 10 mm HEPES (pH 7.4), papain (1000 U mL^−1^), and 1 × DNase I (250 U mL^−1^)) for 30 min at 37 °C, shaking every 5 min in this period. The papain solution was then replaced with equal volume of Ca^2+^‐ and Mg^2+^‐free HBSS within DNaseI (250 U mL^−1^) and Ovomucoid (Sangon Biotech, A003085, 1:50) to terminate the reaction in a 37 °C incubator for 2 min. Being shortly centrifuged (1000 rpm, 1 min), precipitate was resuspended using 2 mL HBSS and gently pipetted with fire‐polished glass pipettes for six to eight times, until all tissues were dissociated into single cells. After several round of short centrifugation (400 rpm for 1 min) and pipetting (using decreasing bore diameter of fire‐polished glass pipettes each round). The suspension was filtered via 70 µm cell strainers, subsequently centrifuged (1600 rpm, 8 min) to collect single cell pellets and resuspended in cold PBS for further studies.

##### Fluorescence‐Activated Cell Sorting

Single cell pellets were passed through cell strainer (35 µm). GFP or Tom‐positive cells were sorted by FACS (MoFloAstrios EQS, Beckman Coulter). FACS gating strategy for sorts was shown in Figure S1H,I, Supporting Information. SSC‐H and FSC‐H discrimination was used for excluding cell debris, while doublet cells were excluding by FSC‐W and FSC‐H/A discrimination.

##### Tissue and Immunofluorescence

Mice at defined ages were sacrificed through transcardially perfused with ice‐cold PBS following by cold 4% paraformaldehyde (PFA), and brains being fixed at 4 °C overnight in 2% PFA. All samples were sagittally dissected along the midline. For cryo‐sections, brains were embedded in OCT (Leica, 14 020 108 926) and frozen at −80 °C until being serially sectioned at 12 µm using a cryostat (Leica, CM1950). As for paraffin sections, sections were cut at 5 µm thickness and it is necessary for the slides to go through deparaffization and rehydration before microwave heat‐induced antigen retrieval. Slides washed with PBS and blocked with PBS containing 0.4% Triton ×‐100 and 3% normal BSA for 0.5 h at room temperature (RT), and primary antibodies were applied at different dilution in 1% blocking solution overnight at 4 °C. After washing with PBS, sections were incubated with secondary antibodies conjugated to Cy2, Cy3, or Cy5 mixed with DAPI for 1 h at RT or 3 h at 4 °C. Slides were washed again with PBS, covered with mounting medium and coverslipped. Images were captured with a confocal laser microscope (LEICA, SP8).

##### Histological Analysis

Paraffin sections from histologically comparable positions of control and Setdb1^mGFAP^CKO cerebella at P15 were stained by H&E. After deparaffinage and rehydration, slides were soaked in Hematoxylin (5 min) and Eosin Y (≈30 s). Slides with appropriate staining intensity went through dehydration and mounted in neutral balsam for further image capture with OLYMPUS CX23 microscope (ISH500).

##### X‐Gal Staining

Frozen sections from P15 mGFAP‐Cre, R26‐stop‐LacZ mice were subjected to X‐gal staining for 1 h to overnight according to the signal intensity, and the images were captured by OLYMPUS CX23 microscope (ISH500).

##### Proximity Ligation Assay

Sagittal cryo‐sections used in the PLA study were fixed shortly for 1 h in 2% PFA. The procedures followed previous report with little modified.^[^ ^55^ ^]^ In brief, mixing the components well prior to usage in each step, slides were incubated with blocking solution for 1 h at 37 °C before being taken off. Anti‐Trim28, anti‐Nfix, and anti‐IgG antibodies mixing with anti‐GFP and anti‐Setdb1 were diluted by the Duolink antibody diluent solutions and added to each brain sample. Slides were then incubated in a humidity chamber overnight at 4 °C. After being washed 3 × 5 min in 1 × wash buffer A at RT, the PLUS and MINUS PLA probes diluted in Duolink antibody diluent were then added to the slides subsequently incubating in a pre‐heated humidity chamber for 1 h at 37 °C. In the following two steps, slides were washed again with wash buffer A before being incubated with the ligation solution for 30 min at 37 °C and amplification solution for 100 min at 37 °C. Washing three times in 1 × wash buffer B for 2 × 10 min at RT, slides were incubated with secondary antibodies and DAPI for 30 min at 37 °C. Finally, washing twice with 1 × wash buffer B for every 10 min following by 0.01 × wash buffer B one time, slides were coverslipped by Duolink PLA mounting medium. Slides can be stored in the dark at 4 °C for up to 4 days before imaging till being captured with a confocal laser microscope (LEICA, SP8).

##### TMX Administration

TMX was dissolved in sunflower seed oil at a concentration of 20 mg mL^−1^ and stored at −20 °C. For Mash1^iKO^ mice and the control were treated with TMX by subcutaneous injection once a day per mice at a dosage of 50 µg/g (gram, body weight) at P4 and 70 µg/g at P5. While for young adult Mash1^iTom^ mice were gavaged at a dosage of 200 µg/g for three consecutive days.

##### BrdU Assay

BrdU (Roche, 70 164 521) was dissolved in PBS at a concentration of 10 mg mL^−1^. For BrdU chasing GC migration assay, mice were pulsed with 100 µg/g (gram, body weight) of BrdU through intraperitoneal injection at 1 d or 3 d before sacrificed.

##### Ex vivo CSC

This experiment was carried out as previously described, but was changed slightly based on this.^[^ ^23^ ^]^ At first, the P7 mice was intraperitoneally injected with BrdU (100 µg/g), after 39 h, cerebella were dissected, and embedded in 4% low‐melting‐point agarose and 350‐µm sagittal cerebellar slices were prepared using a VT1000S Vibratome (Leica Microsystems). Slices were transferred to Millicell tissue culture inserts (Millipore) and incubated in the culture media (basal Eagle medium supplemented with 2 mm L‐glutamine, 0.5% glucose, 50 U mL^−1^ penicillin‐streptomycin, 1 × B27 (Thermo Fisher SCIENTIFIC), 1 × N2 (Invitrogen), 10 ng mL^−1^ BDNF, 10 ng mL^−1^ GDNF, 1 µm cAMP, 0.55 µm *β*‐ME, and 40 µg mL^−1^ mino) and then cultured for 30 h. To measure the migration distance of GCs, cerebellar slices were fixed with 4% PFA and followed by 15% sucrose and 23% sucrose for dehydration. And then the slices were embedded in OCT for frozen section using Leica, 14 020 108 926), cutting the 350 µm cerebellar slices into 12 µm cerebellar slices. Finally, immunofluorescence staining was performed and the BrdU positive cells in each cerebellar layer were counted.

##### Plasmid Preparation

Mouse Nfix (gene ID: 18 032), human NFIX (gene ID: 4784), and mouse Setdb1 (gene ID: 9869) cDNAs were cloned into insect cell expression vectors, pcDNA3.3‐Flag, and pcDNA3.3‐HA, which in frame with a Flag or HA tag at the N‐terminus.

##### Immunoprecipitation

Human embryonic kidney (HEK293T) cells were cultured in DMEM (Life Technologies, 12800‐082) containing 10% fetal bovine serum (Gibco, 10270‐106), and then plasmids were transfected into them with polyethylenimine (10 µm). Transfected 293T Cells and isolated endogenous BG were homogenized in IP buffer containing 50 mm Tris‐HCl pH 7.4, 150 mm NaCl, 1 mm EDTA, 1 m EGTA, 2.5 mm Sodium Pyrophosphate, 1 mm Sodium orthovanadate and 1% Triton ×‐100, with PMSF (BBI Life Sciences, 329 986, 1:100) and Cocktail (Roche, 1:100) in solution well prior to usage on ice. Ultrasonication was subsequently applied to the cell lysates. After centrifugation, 1 µg anti‐HA or anti‐Flag was added to the supernatant overnight with gentle rotation, then 10 µl protein A/G agarose resin were added and rotate at 4 °C for 3 h. After washed by IP buffer, beads were boiled by 1.2 × SDS sample buffer. Empty vector or anti‐IgG were used as control.

##### Western Blotting

IP samples were separated by 9% SDS‐PAGE and transferred onto PVDF membranes (Millipore). The blots were then blocked in 5% nonfat milk in TBST, following by incubation of primary antibodies at 4 °C overnight. After washing three times, the blots were incubated in Rabbit or mouse horseradish peroxidase‐conjugated secondary antibodies at RT for 1 h or 4 °C for 3 h. Membranes were washed with TBST for three times and imaged using a Bio‐Rad ChemiDoc Touch.

##### MS Sample Preparation and Experiments

For MS analyses, anti‐Flag (20 µl M2 Magnetic Beads, no. M8823) IP were performed with whole‐cell lysates derived from each 10‐cm dishes of HEK293T cells transfected with Flag‐hNFIX, Flag‐mNfix, or Flag‐vector. The IP product were separated by SDS‐PAGE for around 3 cm, stained by Coomassie Blue and excised for MS analysis. All subsequent MS steps were performed at the Core Facility of Biomedical, Xiamen University, and the Peptide sequences by Bruker timsTOF Pro. The number of unique peptides in each protein were drawn into point plot by ggplot2.

##### qRT‐PCR

Total RNA was extracted from sorted BG using TRIzol Reagent (Life Technologies, Carlsbad, California, #15 596 018) following the manufacturer's protocols. The purified mRNA was converted to cDNA with the first‐strand cDNA synthesis kit (ABM) and amplified using the Fast SYBR Green Master Mix (Thermo Fisher). qRT‐PCR was performed using the Bio‐Rad Real‐Time PCR System. Primers used are listed below:

Col18a1‐F: TCTTTTGACGGCAGAGATGTC
Col18a1‐R: AGTTTCAGTTCGCCATGTCTC

##### Rotarod Test

Motor function and balance ability were evaluated by rotarod apparatus. Mice (random sex) were placed into the experimental room 1 h before the rotarod test. The experiment was performed on an accelerating rotarod (Ugo Basile) with 63‐cm fall height, 30‐mm diameter rotating dowel. The rotarod was set for an initial speed of 5 rpm for 30 s and then constantly accelerated from 5 to 40 rpm over 5‐min period. The procedure was repeated over 3 consecutive trials, which was averaged to give the daily latency to fall for each mouse. If an animal fell off the rotarod rapidly (e.g., due to inattention or slips), they were placed back on the rotarod for an additional trial, and the latency was not included in the daily average.

##### Open Field Test

Motor ability including locomotion and spontaneous activity, mouse behavior was characterized as they freely explored an open‐field chamber which is 50‐cm width × 50‐cm length × 40‐cm height. The total distances of one‐month old mice were recorded in this arena for 10 min with a video tracking system (Smart 3.0).

##### RNA‐Seq and Analysis

Total RNA was extracted from sorted BG using TRIzol Reagent (Life Technologies, Carlsbad, California, #15 596 018) following the manufacturer's protocols. RNA was subsequently used for cDNA library construction with RNA integrity numbers up to 7, and then sequencing was performed with about 20 million reads per sample by the BGI Genomics Co., Ltd. using BGISEQ‐500 platform. High‐quality reads were aligned to the mouse reference genome (mm10) using Bowtie2. Expression levels for each of the genes were normalized to expected number of Fragments Per Kilobase of transcript per million base pairs sequenced (FPKM) using RNA‐seq by expectation maximization. DEGs were defined as genes with false discovery rate (FDR) less than 0.001 and fold change larger than 2. The following Pathways and GO analysis were annotated in the KEGG (Kyoto Encyclopedia of Genes and Genomes) database phyper and shown by ggplot2. Heatmap was generated using the pheatmap package and ggplot2 in the R environment.

##### ChIP

Standard ChIP was performed after modification of ChIP‐seq performed as previously described protocol with slight modification.^[^ ^55^ ^]^ Approximately 5 × 10^6^ Bergmann glial cells isolated as above was fixed in 1% formaldehyde for 10 min at RT, followed by quenching in 125 nm glycine for 5 min. Cell nuclei were collected and lysed in lysing buffer (50 mm HEPES/KOH pH 7.6, 1 mm EDTA, 140 nm NaCl, 10% v/v Glycerol, 0.5% NP‐40, 0.25% v/v Triton ×‐100) supplemented with protease inhibitor. Nuclei were subjected to sonication to acquire DNA fragments of 200–350 bp. The sonicated chromatin was applied to IP by incubation with 1.5 µg H3K9me3 antibody (rabbit, Abcam, #ab232324) overnight at 4 °C, followed with collection using 30 µL protein A/G plus agarose beads (Millipore). After elute and purify the chromatin, the ChIP‐seq library was constructed using a KAPA HyperPrep Kits (Roche, 0 796 234 7001) according to the manufacturer's instructions, and then run on the Illumina sequencer Hiseq‐Xten PE150.

##### ChIP‐Seq Data Analysis

The image analysis and base calling were performed by using Illumina's Genome Analysis pipelin. The sequencing reads were aligned to mm10 Refseq database by using Bowtie2 with default parameters. Both uniquely aligned reads and reads that align to repetitive regions were kept for downstream analysis (if a read was aligned to multiple genomic locations, only one location with the best score was chosen). Clonal amplification was circumvented by allowing maximal one tag for each unique genomic position. The identification of ChIP‐seq peaks was performed using HOMER. The threshold for the number of tags that determined a valid peak was selected at a FDR of 0.001. Fourfold more tags relative to the local background region (10 kb) were also required to avoid identifying regions with genomic duplications or non‐localized binding. Genomic distribution was done by using the default parameters from HOMER with minor modifications, in which Annotated positions for promoters, exons, introns, and other features were based on RefSeq transcripts. Motif analysis was performed using HOMER.

##### Statistical Analyses

No statistical methods were used to predetermine sample sizes, and the exact values of *n* (sample size) are provided in the Results section and figure legends. In all cases where an unpaired *t*‐test was employed, a two‐tailed test was opted for. For behavioral analysis, open field test (the motor ability) and rotarod test (the balance ability) were rated and the percentage of observations was calculated for each mouse, and data represent mean ± SEM. During behavioral tests, the data were captured and analyzed using a fully automatic analysis program. All statistical analyses were performed using GraphPad Prism version 7.0, and the data presented are of mean ± SEM. Null hypotheses were rejected at p values equal to or higher than 0.05. The *, **, **** and ns denote *p*< 0.05, *p*< 0.01, *p*< 0.0001 and *p*> 0.05, respectively. For FACS and sequencing, the investigators were blinded to group allocation during data collection and analysis. Quantification of BG numbers in PC‐BG layer. Anatomically comparable sections from control and mutant brains were visualized under 20× magnification using a Leica SP8 microscope. Quantifications were performed from at least three groups of mice in a blinded fashion. Lengths, areas, and the number of cells were quantified using the ImageJ software package.

## Conflict of Interest

The authors declare no conflict of interest.

## Author Contribution

S.C., K.Z., and B.Z. contributed equally to this work. S.C. and W.M. designed experiments, performed data analyses, and wrote the manuscript; S.C., B.Z., Y.X., B.J., Y.G., Y.Y., T.Q., and K.Z. performed experiments; S.C., S.L., and M.J. data visualization; X.Z., Y.G., K.Z., Q.C., Z.C., H.L., and J.H. discussed and edited the manuscript; W.M. supervised the project. All authors reviewed and gave final approval to the manuscript.

## Supporting information

Supporting Information

Supplemental Table 1

## Data Availability

All data and code used to generate the data are available upon reasonable request to the authors.
