# Supplementary material for: Temporarily Epigenetic Repression in Bergmann Glia Regulates the Migration of Granule Cells
Source: Adv Sci (Weinh). 2021 Mar 22;8(10):2003164. doi: 10.1002/advs.202003164 (PMC8132163; doi:10.1002/advs.202003164)
Supplement: Supplementary file 1 — Supporting Information [file ADVS-8-2003164-s002.pdf]

© 2021 Wiley-VCH GmbH

ADVANCED SCIENCE
Open Access

# Supporting Information

for *Adv. Sci.,* DOI: 10.1002/advs.202003164

# Temporarily epigenetic repression in Bergmann glia regulates the migration of Granule cells

*Shaoxuan Chen, Kunkun Zhang, Boxin Zhang, Mengyun Jiang, Xue Zhang, Yi Guo, Yingying Yu, Tianyu Qin, Hongda Li, Qiang Chen, Zhiyu Cai, Site Luo, Yi Huang, Jin Hu, Wei Mo**

# Supporting Information

**Temporarily epigenetic repression in Bergmann glia regulates the migration of Granule cells**

*Shaoxuan Chen, Kunkun Zhang, Boxin Zhang, Mengyun Jiang, Xue Zhang, Yi Guo, Yingying Yu, Tianyu Qin, Hongda Li, Qiang Chen, Zhiyu Cai, Site Luo, Yi Huang, Jin Hu, Wei Mo**

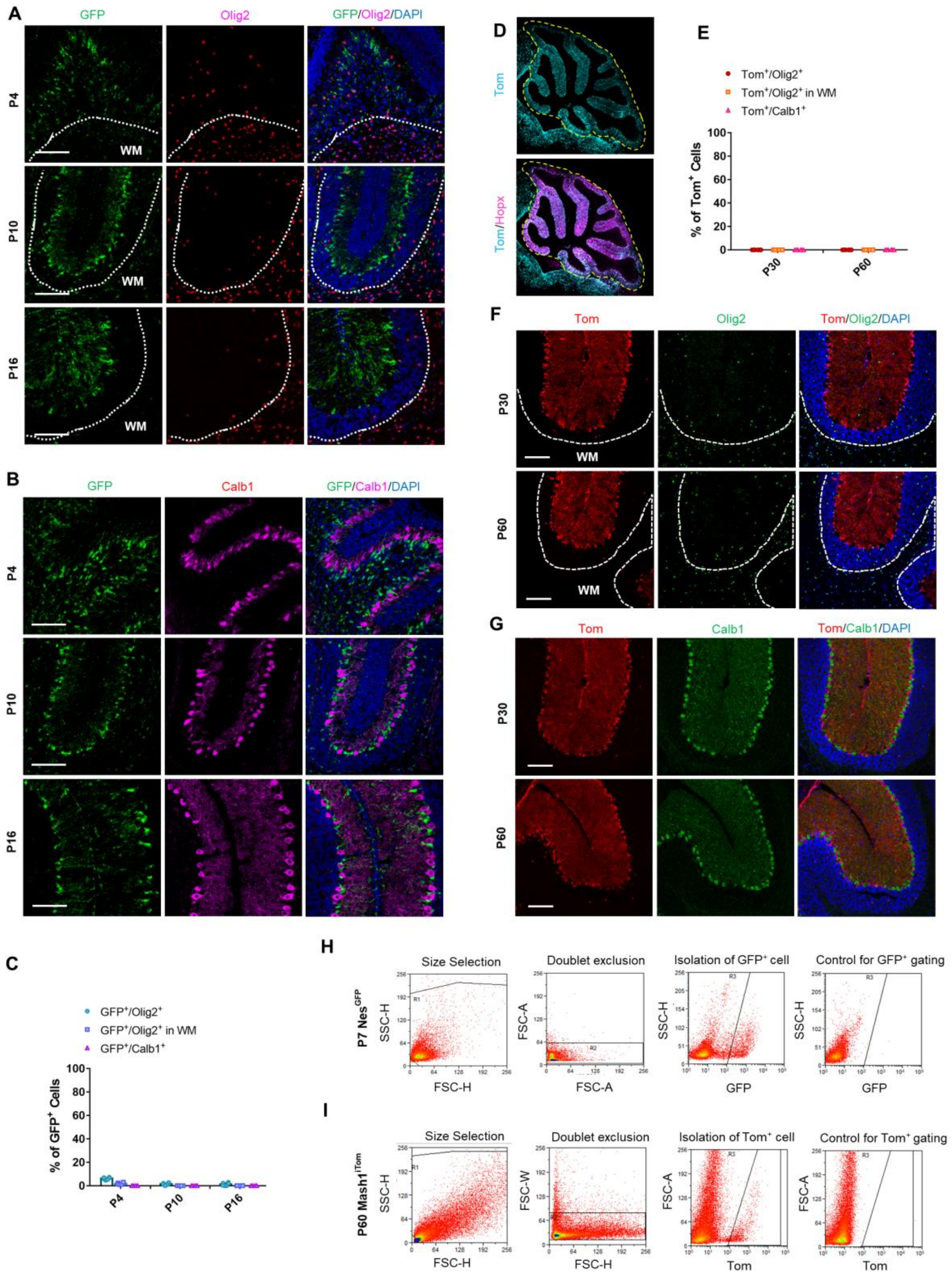


## Supplementary Figure legends

**Figure S1. Verifying off-target of the mouse models and isolating Bergmann glia, Related to Figure 1**

(A-C). Co-immunostaining of GFP with Olig2 (A) or Calbindin (Calb1) (B). The ratio of different kinds of GFP positive cells at each time point (P4, P10, P16) in $Nes^{GFP}$ mice were quantified (C). OLs: $Olig2^{+}$ cells in WM; PCs: $Calb1^{+}$ cells. n = 6 sections from 3 mice per time point. (D). The expression of Tomato and Hopx within the range of whole

1-month-age cerebellum (in the white line region) to illustrate the specify of the Mash1$^{iTom}$ mice. (E-G). Co-immunostaining of Tom with Olig2 (F) or Calbindin (Calb1) (G). The ratio of different kinds of Tom positive cells in Mash1$^{iTom}$ mice at time points as indicated were quantified (E), OLs: Olig2$^{+}$ cells in WM; PCs: Calb1$^{+}$ cells. n = 6 sections from 3 mice per time point. (H-I). The example of gating GFP$^{+}$ BG at juvenile stages with Nes$^{GFP}$ mice or Tom$^{+}$ BG at young adult stages with Mash1$^{iTom}$ mice. The wildtype cerebella at the same time point were used for negative control. All the quantification data are presented as mean ± SEM. Scale bars, 100 μm.

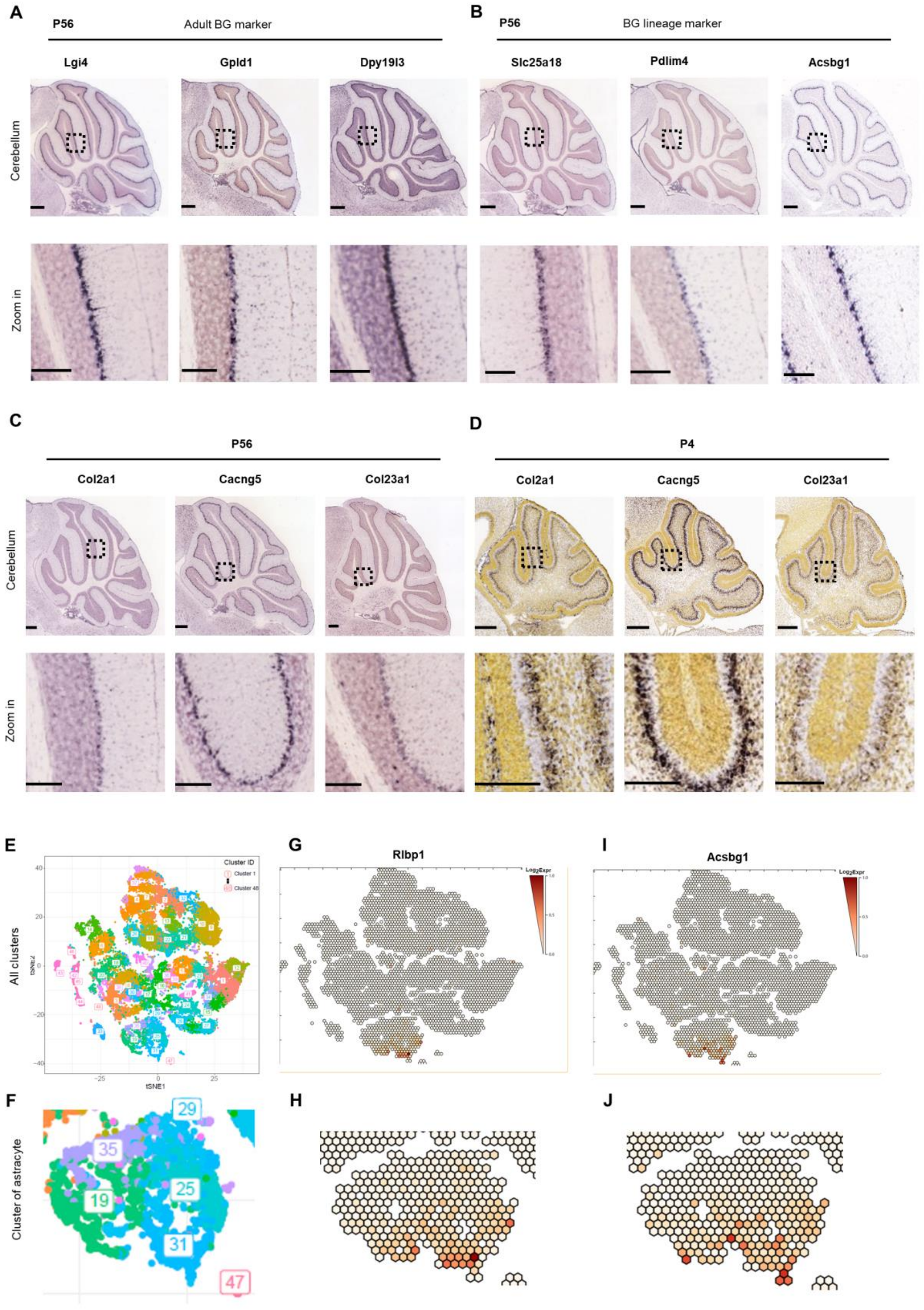


**Figure S2. Compare the Bergmann glia new markers to public database**

(A, B). In situ hybridization staining for the examples (related to figure 2C) of new adult BG markers (A) and BG lineage markers (B) in P56 cerebellum and local enlarged drawing (data obtained from the Allen Brain Atlas). (C, D). In situ hybridization staining for some selected BG lineage markers with moderate expression (10<FPKM<50) in P56 (C) and P4 (D) cerebellum and local enlarged drawing (data

obtained from the Allen Brain Atlas). FPKM, fragments per kilobase of transcript per million mapped reads. Scale bars, 400 μm. (E-I). Previous study (ref.[36]) shows the two-dimensional visualization of single-cell clusters using t-SNE (E). Zoom-in images indicated the cluster of astrocytes, of which the Bergmann glia in clusters 19 and 31. The expression of BG new marker genes was checked in “Cell Seek” website where the subpopulations were shown. Rlbp1 and Acsbg1 were expressed in BG populations (G, I), and the corresponding zoom-in images (H, J).

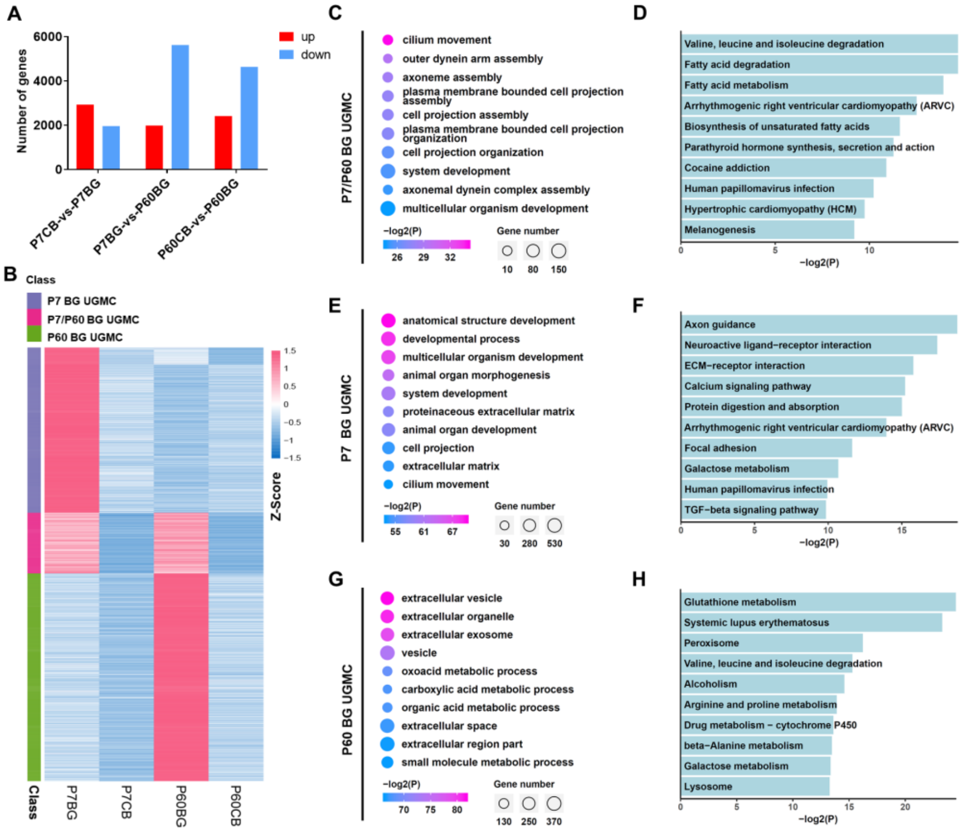


**Figure S3. Functional analysis for UGMC of Bergmann glia**

(A). The histogram shows DEGs (differentially expressed genes, fold change≥2,) of BG vs other type of cells in cerebella at the same time point and DEGs between juvenile and young adult BG. (B). Heatmap of UGMC as indicated. (C-H). GO and Pathway analysis on UGMC listed in (B). P7/P60 (C, D), P7 (E, F) and P60 (G, H). Data show the top 10 enriched terms ranked by p values. For GO, the color indicates p values for each term GO enrichment and the circle size indicates the number of enriched genes within each GO term. For Pathway, the p values of the enriched pathway in accordance with the abscissa of bar charts.

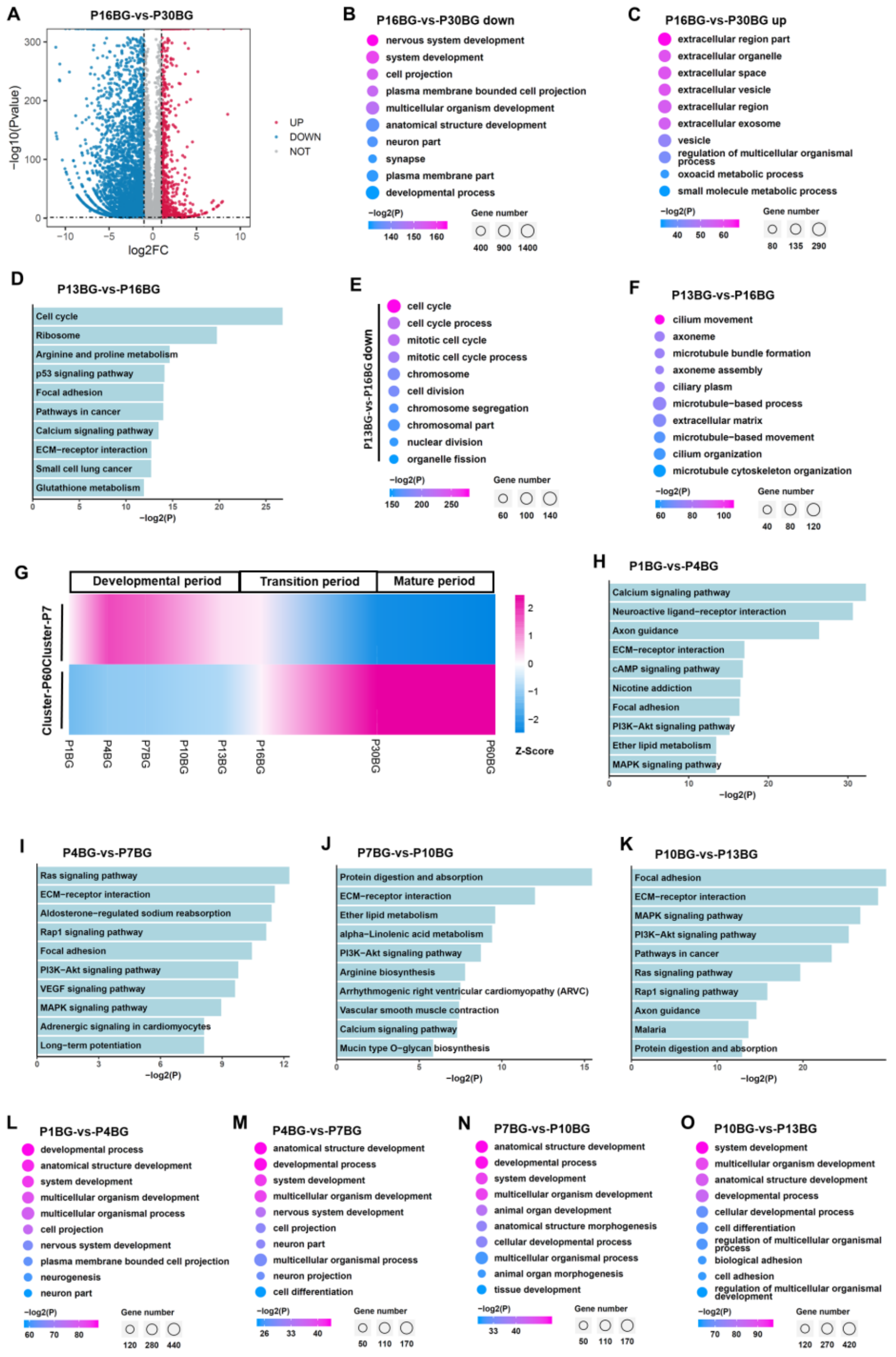

**Figure S4. Functional analysis for Bergmann glia DEGs in each stage of development**

(A). Volcano plot shows the DEGs between P16 BG and P30 BG. (B, C). Representative top10 GO terms enriched from downregulated (B) or upregulated (C) DEGs of adjacent stage (P16 BG versus P30 BG). (D). Representative top10 pathway terms enriched in DEGs of adjacent stage (P13 BG versus P16 BG). (E, F). Representative top10 GO

terms enriched from downregulated (E) or total (F) DEGs of adjacent stage (P13 BG versus P16 BG). (G). Heatmap claims 3 period of BG from birth to young adult. Cluster-P7 is the average expression from P7 BG UGMC and cluster-P60 were the average expression from P60 BG UGMC (related to Figure S3B, Supporting Information). (H-O). Representative top10 Pathway terms (H-K) and GO terms (L-O) enriched from DEGs of each coterminous stage during first 2 weeks postnatally. All data display top 10 enriched GO or Pathway terms ranked by p values.

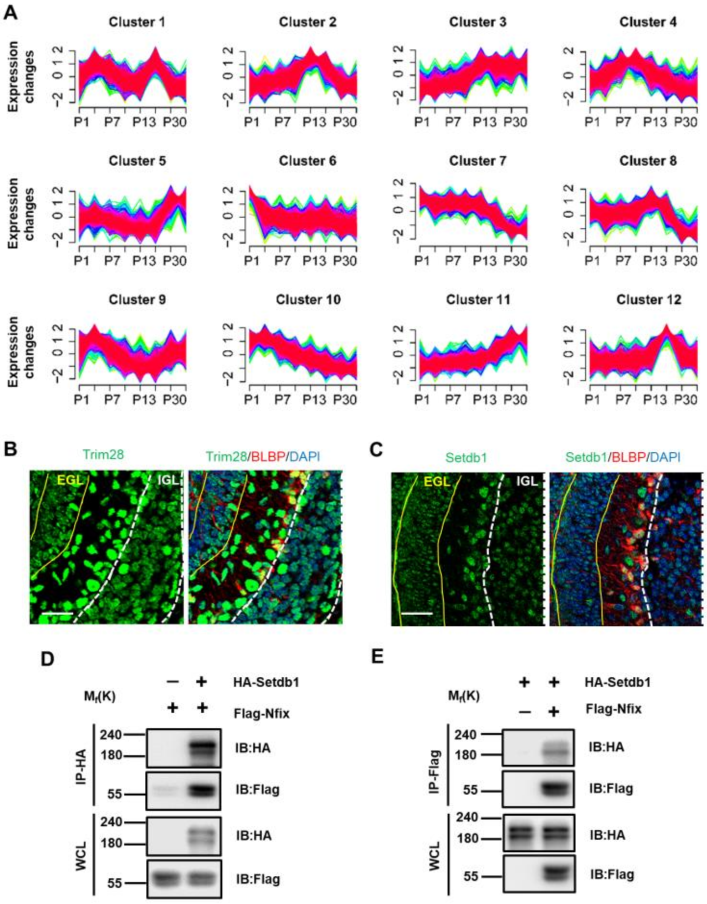


**Figure S5. Identification of Nfix-Setdb1 repressive complex for transcriptional repression in BG from P7 to P10, Related to Figure 4**

(A). Time series analysis shows all the transcripts of BG in different developmental time points from P1 to P30. Different clusters represent gene sets with similar expression pattern during BG development, and all these genes are divided into 12 clusters. (B, C). Immunofluorescence staining shows the expression of Trim28 (B) and Setdb1 (C) in P10 BG. Scale bars, 30 μm. (D, E). Immunoblot (IB) analysis of whole cell lysates (WCL) and immunoprecipitation (IP) products derived from 293T cells transfected with indicated plasmids.

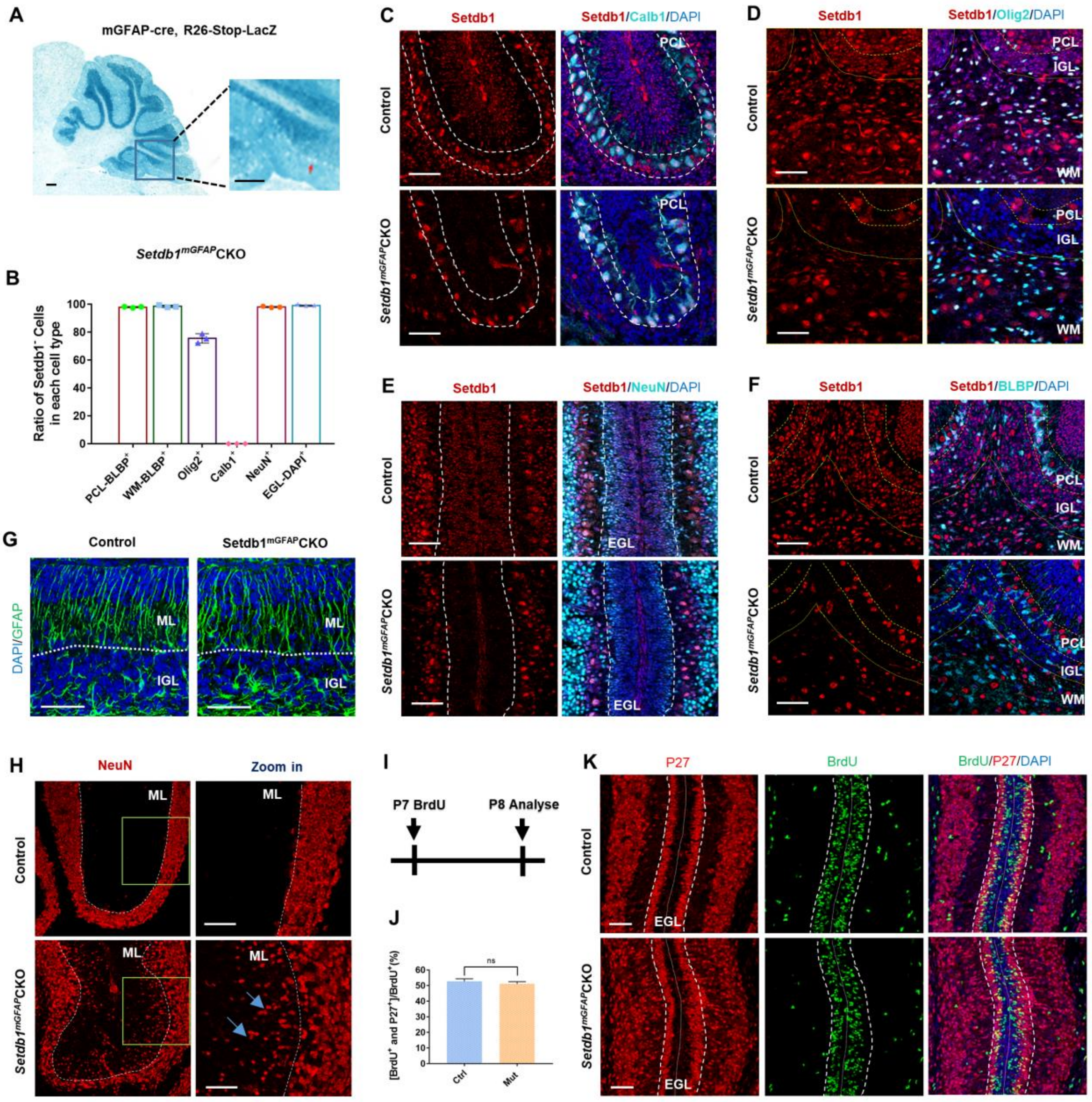


**Figure S6. Setdb1 deficiency of Bergmann glia restrains GCs migration, Related to Figure 5**

(A). X-gal staining of brain sections from P15 mGFAP-Cre, R26R-LacZ mice. Arrow points to the Purkinje cells which are not targeted by mGFAP-Cre. (B-F). Comparison of the ratio of Setdb1$^{-}$ cells within different types of cells in *Setdb1*$^{mGFAP}$CKO (Mut) mice (n = 3) (B), and their corresponding staining (C-F). Almost completely knockout *Setdb1* in BG (PCL-BLBP$^{+}$), astrocyte (WM-BLBP$^{+}$), GCs (NeuN$^{+}$ and EGL-DAPI) and Olig2$^{+}$ OLs. (G). Immunostaining for GFAP in the control and *Setdb1*$^{mGFAP}$CKO mice at P7. (H). NeuN staining shows the mature GCs in the control and

*Setdb1*$^{mGFAP}$CKO cerebella at P30. Arrows point to the ectopic cell mass in the ML in mutant mice. (I-K). 1-day BrdU pulse-chase assay was performed (I). Cerebellar sections from P8 control and *Setdb1*$^{mGFAP}$CKO mice were stained for P27/BrdU (K). The BrdU and P27 double positive cells in the total BrdU$^{+}$ cells were quantified, n = 22 sections from 3 mice for each group (J). ns, no significance. All the quantification data are presented as mean ± SEM, two-tailed unpaired Student's t-test. Scale bars, 50 µm.

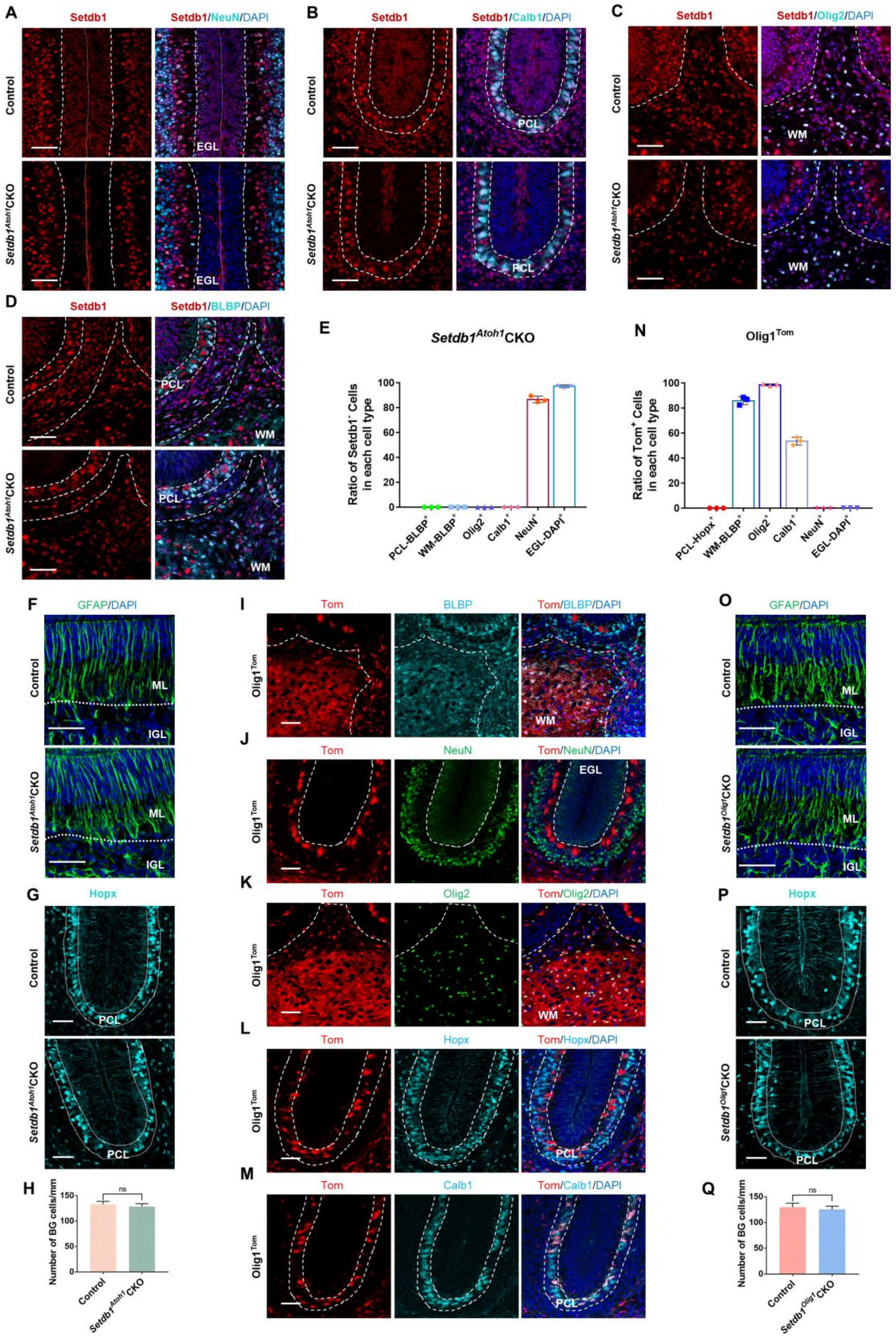


**Figure S7. The absence of Setdb1 in GNPs or OLs has no major effects on BG**

(A-E). Identify the knock out efficiency of Atoh1-Cre by co-immunostaining of Setdb1 with NeuN (A), Calb1 (B), Olig2 (C) and BLBP (D) in *Setdb1*$^{Atoh1}$CKO and control mice. The ratio of Setdb1 knock out (E) in GC (EGL-DAPI$^{+}$ and NeuN$^{+}$), OL (Olig2$^{+}$), Astrocyte (WM-BLBP$^{+}$), BG (PCL-BLBP$^{+}$) and PC (Calb1$^{+}$) at P7 was statistically

analyzed (n = 3). (F-H). Staining of GFAP (F) and Hopx (G) in the control and *Setdb1*$^{Atoh1}$CKO cerebella at P7 (BG cell bodies were in the PCL), and the density of cell bodies of BG in PCL was quantified (H). ns, no significance, n = 6 sections from 2 mice for each group. (I-N). Co-immunostaining of Tom with BLBP (I), NeuN (J), Olig2 (K), Hopx (L), and Calb1 (M) in Olig1$^{Tom}$ mice. The ratio of Tom$^+$ cells (N) in GC (EGL-DAPI$^+$ and NeuN$^+$), OL (Olig2$^+$), Astrocyte (WM-BLBP$^+$), BG (PCL-Hopx$^+$) and PC (Calb1$^+$) at P7 was statistically analyzed (n = 3). (O-Q). Staining of GFAP (O) and Hopx (P) in the control and *Setdb1*$^{Olig1}$CKO cerebella at P7, and the density for cell bodies of BG in PCL was quantified (Q). ns, no significance, n = 6 sections from 2 mice for each group. All the quantification data are presented as mean ± SEM, two-tailed unpaired Student's t-test. Scale bars, 50 μm.

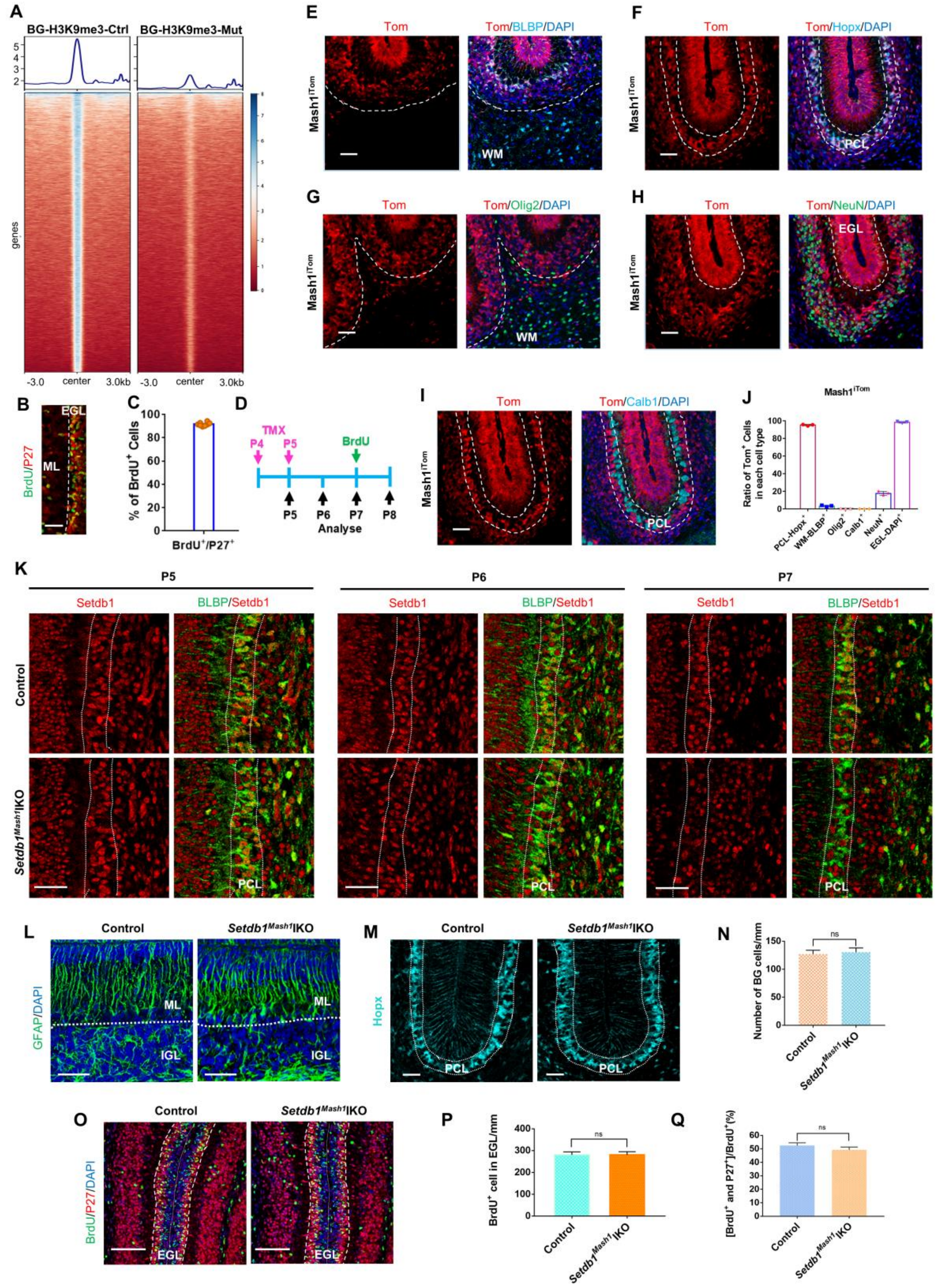


**Figure S8. Nfix-Setdb1 transcriptional inhibitory complex targets genes that downregulated for GC migration during P7-P10, Related to Figure 6**

(A). Heatmap shows Setdb1-mediated H3K9me3-binding sites. ±3 kb surrounding H3K9me3-binding summits. (B-C) P27 staining for the BrdU$^{+}$/GC (B) indicates over 90% of these cells (C) are out of cell cycle (n = 8 sections from 2 mice). (D). Workflow

of TMX administration in *Setdb1*$^{Mash1}$IKO mice and BrdU pulse-chase assay. (E-J). Staining (E-I) and analysis of the ratio of Tom$^+$ cells within different types of cell (J) in Mash1$^{iTom}$ mice at P7 (n = 3). Nearly 100 % of BG (PCL-Hopx$^+$) and GNPs (EGL-DAPI$^+$) are Tom$^+$ cells, whereas a few of Tom$^+$ cells in OL (Olig2$^+$), Astrocyte (WM-BLBP$^+$), BG (PCL-Hopx$^+$) or PC (Calb1$^+$). (K). The residue of Setdb1 in BG of *Setdb1*$^{Mash1}$IKO mice upon TMX administration. The cell body of BG cells are located between the dash lines. (L-N). Staining of GFAP (L) and Hopx (M) in the control and *Setdb1*$^{Mash1}$IKO cerebella at P7, and the density of BG bodies in PCL (N) was quantified (ns, no significance, n = 6 sections from 2 mice for each group. (O-Q). P8 cerebellar sections from control and *Setdb1*$^{Mash1}$IKO mice were stained for P27/BrdU (O). Density of BrdU$^+$ cells in EGL (P) and the ratio of BrdU$^+$ and P27$^+$ cells in the total BrdU$^+$ cells (Q) was quantified, n = 12 sections from 3 mice for each group, 24h after BrdU-pulse. ns, no significance. All the quantification data are presented as mean ± SEM, two-tailed unpaired Student's t-test. Scale bars, 40 μm.

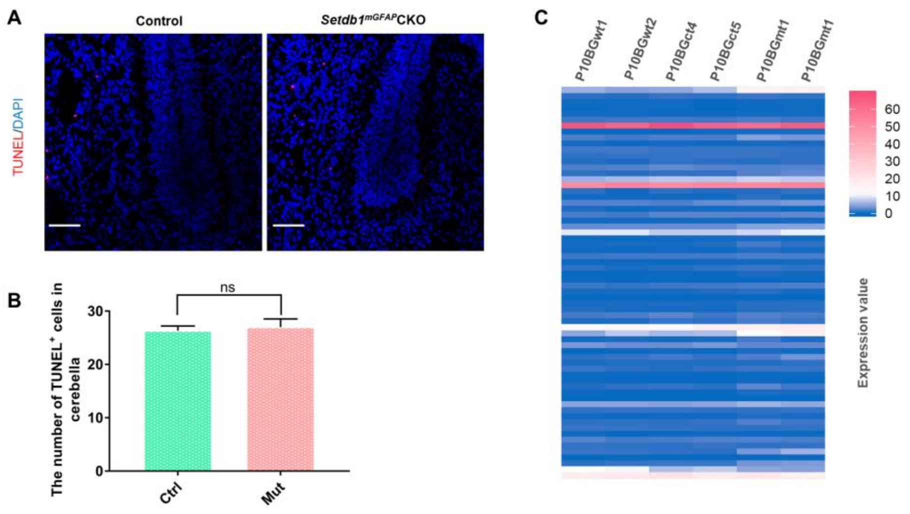


**Figure S9. Neither significant cell death nor inflammation was detected in *Setdb1* deleted BG**

(A, B). TUNEL staining of P7 cerebella (A) and the TUNEL$^+$ cells in cerebella were quantified, n = 3 from 3 mice for each group (B). (C). Heatmap shows the expression of inflammatory genes (ref.[28]) in the indicated samples. ns, no significance.
